# Tucum‐do‐Cerrado (*Bactris setosa* Mart.) Consumption Promoted a Healthier Expansion of Adipose Tissue in High‐Fat Diet‐Induced Obesity Rats

**DOI:** 10.1002/mnfr.70498

**Published:** 2026-06-30

**Authors:** Marilia Hermes Cavalcanti, Amílcar Sabino Damazo, Sandra Fernandes Arruda

**Affiliations:** ^1^ Postgraduate Program in Human Nutrition Faculty of Health Sciences Campus Universitário Darcy Ribeiro Universidade de Brasília Brasília Brazil; ^2^ Postgraduate Program in Tropical Medicine Faculty of Medicine Universidade de Brasília Brasília Brazil

**Keywords:** adipose tissue, high‐fat diet, lipid metabolism, redox, Tucum‐do‐Cerrado

## Abstract

Tucum‐do‐Cerrado (*Bactris setosa Mart*.) is a polyphenol‐rich Brazilian fruit known to improve glucose metabolism. This study investigated its effects on brown (BAT), inguinal (iWAT), and epididymal (eWAT) adipose tissues in a diet‐induced obesity model. Rats received control (CT/TUC‐), high‐fat (HF/TUC‐), control with Tucum‐do‐Cerrado (CT/TUC+), or high‐fat with Tucum‐do‐Cerrado (HF/TUC+) diets. Tucum‐do‐Cerrado promoted healthier adipose expansion by stimulating adipogenic/thermogenic‐related genes while downregulating lipogenesis. In BAT, HF/TUC+ attenuated adipocyte hypertrophy and decreased the number of unilocular adipocytes. Tucum‐do‐Cerrado downregulated *Fasn* mRNA levels and glutathione peroxidase (GPx) activity, whereas it increased thermogenesis‐related *Ucp1* mRNA. In the iWAT, HF/TUC+ decreased the adipocyte area and oxidative markers, while increasing the number of multilocular adipocytes and UCP1 protein levels. Tucum‐do‐Cerrado decreased *Acaca, Prkaa2*, *Il10* mRNA, and GPx activity; conversely, it stimulated the thermogenesis‐related genes *Ppargc1a* and *Prdm16*. Regarding eWAT, its consumption increased the number of multilocular adipocytes, *Prkaa1, Ppargc1a, Ucp1*, *Prdm16*, and *Vegfa* mRNA levels, carbonyl levels, and GPx activity, while decreasing *Acaca* and *Fasn* mRNA levels. Therefore, Tucum‐do‐Cerrado prevented adipocyte hypertrophy and increased the number of multilocular adipocytes. The beneficial effects of Tucum‐do‐Cerrado in an obesity model may be attributed to enhanced antioxidant capacity and inflammatory status, highlighting its potential in attenuating adipose tissue dysfunction.

## Graphical abstract

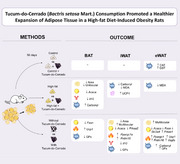

**Abbreviations**
ANOVA: analysis of variance
ATGL: adipose triglyceride lipase
BAT: brown adipose tissue;
CAT: catalase
CT/TUC‐: control diet
CT/TUC+: control diet with Tucum‐do‐Cerrado
eWAT: epididymal white adipose tissue
GPx: glutathione peroxidase
GST: glutathione‐S‐transferase
HF/TUC‐: high‐fat diet
HF/TUC+: high‐fat diet with Tucum‐do‐Cerrado
iWAT: inguinal white adipose tissue
MDA: malonaldehyde
mRNA: messenger RNA
NAFLD: non‐alcoholic fatty liver disease
PGC‐1α: peroxisome proliferator‐activated receptor‐gamma coactivator‐1alpha
PPARγ: peroxisome proliferator‐activated receptor gamma
PRDM16: PR domain containing 16
ROS: reactive oxygen species
TUC‐: without Tucum‐do‐Cerrado
TUC+: with Tucum‐do‐Cerrado
UCP1: uncoupling protein 1
WAT: white adipose tissue.

## 1 Introduction

Obesity is one of the most significant public health challenges of the century, affecting millions of individuals worldwide. A high‐fat diet is recognized as one of the main etiological factors in its development and complications, contributing to energy and metabolic imbalances due to excessive body fat accumulation [1]. As obesity is associated with several high‐morbidity and mortality metabolic comorbidities [2], the search for new dietary strategies for the prevention and treatment of obesity has become a research priority.

Adipose tissue, traditionally regarded as a mere lipid storage depot, is now recognized as a dynamic endocrine organ that plays a pivotal role in metabolic homeostasis [3]. One of the roles of this tissue is to regulate triglyceride anabolism and catabolism (lipogenesis and lipolysis), glucose metabolism, and thermogenesis (dissipation of energy as heat). There are two main types of adipose tissue: white adipose tissue (WAT), which specializes in storing energy as triglycerides, and brown adipose tissue (BAT), which is responsible for non‐shivering thermogenesis. Furthermore, some adipocyte subpopulations in WAT can reversibly differentiate into beige adipocytes under certain stimuli, sharing thermogenic characteristics with brown adipocytes [4].

The activation and recruitment of brown adipocytes, as well as the differentiation of white adipocytes into beige adipocytes expressing thermogenic‐associated markers, are promising therapeutic targets for increasing energy expenditure and treating obesity [5]. Their ability to uncouple the electron transport chain to dissipate energy as heat within the mitochondria increases oxidative metabolism and influences systemic lipid and glucose homeostasis, which is associated with improved plasma lipid profiles [6, 7]. Adipose tissue thermogenesis is orchestrated by a complex network of molecular regulators. PRDM16 (PR domain containing 16), a key transcription factor common to both brown and beige adipocytes, and Peroxisome Proliferator‐Activated Receptor Gamma Coactivator 1‐alpha (PGC‐1α), a master transcriptional coactivator of mitochondrial biogenesis and thermogenesis, are among the most studied regulators that increase thermogenesis by increasing the transcription of Uncoupling Protein 1 (UCP1) [7, 8]. The increase in lipolysis from lipid droplets also contributes to an increase in UCP1 activity and thermogenesis.

The consumption of fruits rich in polyphenols has been associated with a reduced risk of obesity, adipocyte browning, and induced thermogenesis, especially those found in purple fruits [9]. Jaboticaba peel extract reduced energy intake, weight gain, fasting glucose, dyslipidemia, and prevented the nonalcoholic fatty liver disease in mice, by modulating lipid metabolism and inflammatory response [10]. It has also been shown that jaboticaba peel and seed powder reduce weight gain, adipocyte size, and improve glucose metabolism and insulin sensitivity in a high‐fat diet obesity model in mice [11]. Açai seed extract, a source of proanthocyanidins, inhibited pre‐adipocyte differentiation and suppressed adipogenesis in 3T3‐L1 adipocytes, in addition to reducing weight gain and retroperitoneal adipocyte area in mice treated with a high‐fat diet [12].

Tucum‐do‐Cerrado (*Bactris setosa* Mart.) is a palm species native to the Brazilian Cerrado biome, and its purple fruits are a source of polyphenols with high antioxidant potential [13]. Previous studies have demonstrated that Tucum‐do‐Cerrado increases glucose uptake and oxidation in the liver and muscle of obese rats, thereby improving glucose metabolism [14]. Additionally, in a rat model of oxidative stress induced by excess iron, Tucum‐do‐Cerrado inhibited the gluconeogenic rate‐limiting enzyme, glucose 6‐phosphatase, and upregulated glucose transporter 2 hepatic glucose uptake, improving glucose metabolism [15].

Given the strong relationship between glucose and lipid metabolism, this study investigated the effects of Tucum‐do‐Cerrado consumption on the metabolism and adiposity of inguinal adipose tissue (iWAT), epididymal adipose tissue (eWAT), and BAT in a diet‐induced obesity model, as well as their interrelationship with inflammatory‐redox responses.

## 2 Materials and Methods

### 2.1 Preparation of the Tucum‐do‐Cerrado Pulp and Peel

The Tucum‐do‐Cerrado (*Bactris setosa* Mart.) fruits were obtained from the state of Goiás during the fruiting period, between January and March (16°28'15.4'' S and 49°03'44.1'' W, Terezópolis de Goiás, Brazil). The fruits were washed with distilled water and stored at −80°C. Subsequently, the seeds were extracted and discarded from the frozen fruits in a dark location shielded from light. The pulp and peel were homogenized, frozen at ‐80°C, and freeze‐dried for further analysis.

The quantity of Tucum‐do‐Cerrado (*Bactris setosa* Mart.) that was added to the diets was 150 g/kg diet. This concentration was determined based on the recommended daily fruit consumption for adults [16] and previous studies conducted by our research group. For a 60 kg human, based on body surface area conversion [17], this equates to a daily intake of approximately 90 g of fresh fruit, which corresponds to one standard fruit serving. After freeze‐drying, 150 g of Tucum‐do‐Cerrado pulp and peel yielded 28 g of freeze‐dried fruit powder. Therefore, 28 g of freeze‐dried Tucum‐do‐Cerrado pulp and peel was added per kg of diet, and the nutritional composition of the fruit and experimental diets is provided in Supplementary Table S1. The Tucum‐do‐Cerrado are previously characterized by a high content of bioactive compounds, including total flavanols (717 mg catechin eq./100 g), anthocyanins (83 mg/100 g), and yellow flavonoids (42 mg/100 g), as well as Vitamin C (78 mg/100 g) [18].

### 2.2 Animals and Diet

Twenty‐eight male Wistar rats, newly weaned (21 days old), purchased from the Instituto de Ciências Biomédicas, USP, São Paulo, Brazil, were individually housed in stainless‐steel cages in a room with a 12/12 h light/dark cycle and a temperature of 22 ± 1°C. The rats had ad libitum access to water, and their diet was provided daily from 12:00 PM to 8:00 AM. The experimental procedures were ethically approved by the Animal Use Ethics Committee (CEUA) of the University of Brasília/UnB under Protocol No. 25/2018 on May 8, 2018, in accordance with the guidelines set by the National Council for the Control of Animal Experimentation (CONCEA) and the “Guide for the Care and Use of Laboratory Animals” of the National Research Council of the National Academies (United States) [19]. Humane endpoints were defined according to the guidelines of the National Centre for the Replacement, Refinement and Reduction of Animals in Research (NC3Rs, United Kingdom) [20]. This study was conducted in compliance with the ARRIVE guidelines [21].

Following a 7‐day acclimatization period during which the rats were fed the AIN‐93G control diet [22], the rats were divided into four experimental groups (*n* = 7/group) and treated for 56 days with one of the following diets: control (CT/TUC‐): AIN‐93G diet; high‐fat (HF/TUC‐): modified control diet containing 58% lipids; Tucum‐do‐Cerrado (CT/TUC+): control diet supplemented with 28 g of freeze‐dried Tucum‐do‐Cerrado/kg diet; high‐fat with Tucum‐do‐Cerrado (HF/TUC+): high‐fat diet containing 58% lipids supplemented with 28 g of freeze‐dried Tucum‐do‐Cerrado/kg of diet. The food ingredients were purchased from Rhoster Brasil (Araçoiaba da Serra, SP, Brazil). The dietary composition is presented in Table S1.

The lipid content of the high‐fat diet (58%) was determined based on the Diet‐Induced Obesity Model from Research 140 Diets, Inc. (#D12492; Research Diets Inc., New Brunswick, NJ, USA). The high‐fat diet contained soybean oil (6%) and lard (52%) as fat sources.

At the end of the treatment period, euthanasia was conducted using 3% isoflurane in an anesthetic chamber, followed by exsanguination through cardiac puncture for blood collection and serum and plasma separation. Immediately after dissection, the liver, interscapular BAT, subcutaneous iWAT, and visceral eWAT were rinsed in saline solution (0.9% NaCl) at 4°C and rapidly frozen in liquid nitrogen (N_2_). Subsequently, all samples were preserved at ‐80°C. A small portion of the liver and each adipose tissue was fixed in 4% formaldehyde solution for histological analysis.

### 2.3 Dietary Intake and Body Weight Changes

The food consumption of the rats was determined daily by calculating the difference between the provided amount (in grams) and the amount remaining for the animals. Weights were measured using a precision scale (Shimadzu, model AUY220, Kyoto, Japan). Weekly, the animals' weight gain was monitored by weighing them on a digital scale (Marte, ASF11, São Paulo, SP, Brazil).

### 2.4 Fecal Energy Excretion

To determine fecal energy excretion, samples from the last week of treatment were dried in an oven at 60°C for 48 h until weight stabilization. The energy content was analyzed using an IKA C2000 basic bomb calorimeter (IKA‐Werke GmbH & Co. KG, Staufen, Germany).

### 2.5 Quantification of Lipid Profile

To evaluate total cholesterol and triglyceride levels in the serum and liver of the animals, commercial kits (BioClin, Belo Horizonte, MG, Brazil) were used according to the manufacturer's guidelines. Lipid extraction from the liver was performed as described by Vieira et al. [23].

### 2.6 Histological Analysis and Immunohistochemistry

All histological experiments were conducted in a blinded manner. Liver and adipose tissue samples were stored in 10% formaldehyde solution. The tissues were then embedded in a buffered solution of 10% formaldehyde and 1% sucrose for 48 h. Following dehydration, the tissues were embedded in paraffin. The tissues were sectioned (5 µm) using a manual microtome (Leica RM2125 RT, São Paulo, Brazil). The resulting slides were stained with hematoxylin and eosin, and three representative images of each section were captured with a 40x objective (scale bar: 10 µm) for WAT and 20x (scale bar: 20 µm) for BAT and liver, using an Axiocam ERc5s camera coupled to a Zeiss Lab.A1 AX10 microscopy (Carl Zeiss Meditec AG, Jena, Germany) sections. To determine the adipocyte area, ImageJ software 1.54 (U. S. National Institutes of Health, Bethesda, Maryland, USA) was used with the Adiposoft 1.16 version (Centro de Pesquisa Médica Aplicada, Pamplona, Espanha) plugin. This program allows for the automatic pick‐up of all adipocytes fully enclosed within the field of view of each image, their labeling with a number, and the automatic measurement of the area of each adipocyte. The identification and counting of multilocular and unilocular adipocytes were performed on three different serial sections of each sample, using a Zeiss Lab.A1 AX10 microscope (Carl Zeiss Meditec AG, Jena, Germany). Images were captured with a 40x objective (scale bar: 10 µm) for BAT unilocular adipocyte, and 100x objective (scale bar: 5 µm) for iWAT and eWAT multilocular adipocytes.

Liver images were evaluated using the Non‐Alcoholic Fatty Liver Disease (NAFLD) score for rodent models by Liang et al. [24].

Immunohistochemistry was used to detect the presence of UCP1 protein in iWAT. The EasyLink Duo kit (EasyPath Diagnósticos, São Paulo, Brazil) was used according to the manufacturer's guidelines. The H‐Score method [25] was used to assess the staining intensity. Image analysis was conducted in a blinded manner.

### 2.7 Gene Expression Analysis (mRNA Levels)

RNA was extracted from adipose tissue according to the manufacturer's instructions using TRIzol reagent (Invitrogen, Carlsbad, CA, USA). RNA integrity was assessed using an electrophoretic profile on a 0.8% agarose gel (BioAgency, São Paulo, SP, Brazil).

Complementary DNA (cDNA) was synthesized using the High‐Capacity cDNA Reverse Transcription Kit with RNase Inhibitor (Applied Biosystems, Foster City, CA, USA) following the manufacturer's instructions. The mRNA levels of the genes listed in Table S2 were quantified using a real‐time polymerase chain reaction system (qPCR; StepOne Plus Fast Real‐Time PCR System, Applied Biosystems, Singapore). The reaction was performed with 2 µL of cDNA, 5 µL of SYBR mix (Applied Biosystems, Foster City, CA, USA), and 0.2 µmol/L (final concentration) of each primer (Table S2), resulting in a final volume of 10 µL. The gene amplification was performed using 1 cycle at 95° for 2 min, followed by 40 cycles under the following conditions: 95°C for 5 s, 60°C for 30 s. The specificity of each amplified product was confirmed using a dissociation melting curve analysis. Relative expression analysis of each gene was performed using the 2‐ΔΔCT method [26].

### 2.8 Oxidative Damage and Antioxidant Capacity

#### 2.8.1 Protein Carbonylation and Lipid Peroxidation

The carbonylated protein concentration in adipose tissue was determined based on the formation of a complex of carbonyl protein groups and the reagent 2,4‐dinitrophenylhydrazine [27] using the molar extinction coefficient of the protein‐DNPH complex, which is 22.308 mmol/L^−1^ cm^−1^ at a wavelength of 450 nm.

To assess lipid peroxidation in adipose tissues, we employed a method based on the reaction of malondialdehyde (MDA) with thiobarbituric acid, following the procedure outlined by Candan & Tuzmen [28]. The results were expressed as nmol of MDA/mg of total protein, and the total protein concentration was measured using the Hartree method [29].

#### 2.8.2 Antioxidant Enzymes

The activities of catalase (CAT), glutathione peroxidase (GPx), and glutathione‐S‐transferase (GST) were analyzed in adipose tissue homogenates. The specific activities of CAT and GPx were determined using the method described by Joanisse & Storey [30]. GST activity was determined by the formation of a 2,4‐dinitrophenyl‐glutathione complex, as described by Habig & Jakoby [31].

### 2.9 Statistical Methods

Statistical analyses were conducted in R (v.4.5.0), using a linear mixed model approach for a 2 × 2 factorial design (Diet × Tucum‐do‐Cerrado). This statistical approach was selected to accommodate the sample sizes across the experimental groups and to ensure robust parameter estimation. ‘Animal’ was included as a random effect. Model assumptions were verified via residual analysis, including Shapiro–Wilk and Levene's tests to evaluate normality and homogeneity of variances, respectively, and violations were addressed using *log1p* transformation, variance modeling, or robust models. Main effects and interactions were performed using a type III factorial ANOVA to obtain the marginal and conditional coefficients of determination. Adjusted means were compared using Tukey's test, with simple effects analysis applied when the Diet × Tucum‐do‐Cerrado interaction was significant.

Despite an initial sample size of *n* = 7 per group, *n* = 4–6 samples were used for techniques with low variability and high reproducibility to optimize the small amount of tissue available. Graphical data represent the statistical outcomes of the Diet × Tucum‐do‐Cerrado interaction. Data from all four experimental groups (CT/TUC‐, CT/TUC+, HF/TUC‐, and HF/TUC+) are presented. Only statistically significant findings are described in the text, and full data are provided in the figures.

## 3 Results

### 3.1 Physiological Parameters and Lipid Profile

**FIGURE 1:**
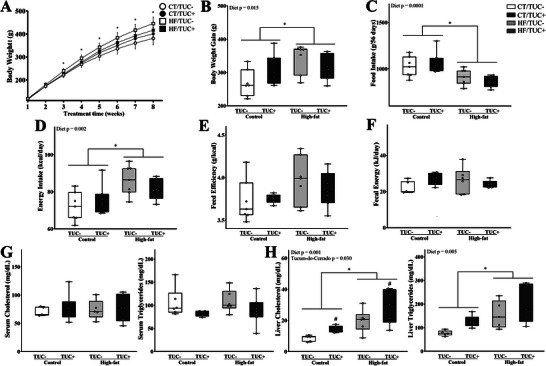
Physiological variables and lipid profile of rats. (A) Body weight gain during the 8‐week follow‐up. (B) Body weight gain. (C) Food intake. (D) Energy intake. (E) Feed efficiency. (F) Fecal energy excretion. (G) Cholesterol and triglycerides in serum and (H) liver. Values are presented as box plots with whiskers from minimum to maximum (*n* = 5–7/group). *p < 0.05 effect of diet; # *p* < 0.05 effect of Tucum‐do‐Cerrado. CT/TUC‐: control diet; CT/TUC+: control diet with tucum‐do‐cerrado; HF/TUC‐: high‐fat diet; HF/TUC+: high‐fat diet with Tucum‐do‐Cerrado; TUC‐: without Tucum‐do‐Cerrado; TUC+: with Tucum‐do‐Cerrado.

Rats fed the high‐fat diet showed increased body weight gain (*p* = 0.015; Figure 1B), energy intake (*p* = 0.002; Figure 1D), and decreased food intake (*p* = 0.0001; Figure 1C), compared to those fed the control diet. The addition of Tucum‐do‐Cerrado did not affect food consumption (Figure 1C), fecal energy excretion (Figure 1F), or weight gain (Figure 1B).

In terms of the lipid profile, the high‐fat diet increased total cholesterol and triglyceride levels in the liver compared with the control diet (*p* = 0.001 and 0.005, respectively; Figure 1H). Tucum‐do‐Cerrado consumption increased the liver cholesterol levels (*p* = 0.030; Figure 1H).

### 3.2 Morphological Characterization of Adipose Tissues

**FIGURE 2:**
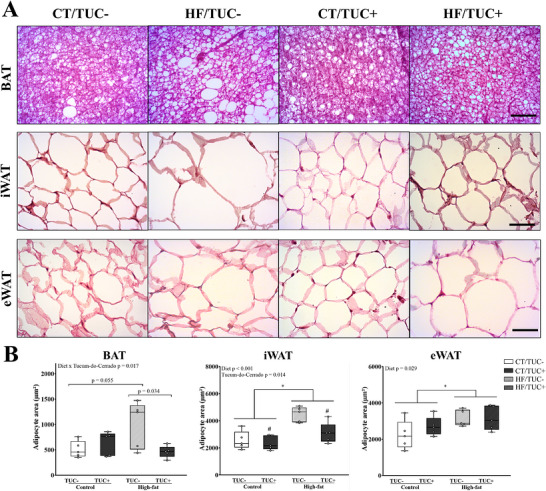
Histological images of adipose tissues of rats. (A) Morphological evaluation of Brown (BAT), inguinal (iWAT), and epididymal (eWAT) adipose tissues. Stain: hematoxylin and eosin. Scale bar: 20 µm (BAT) and 10 µm (iWAT and eWAT) (B) Quantification of unilocular adipocyte area. Values are presented as box plots with whiskers from minimum to maximum (*n* = 5/group). *p < 0.05 effect of diet; # *p* < 0.05 effect of Tucum‐do‐Cerrado. CT/TUC‐: control diet; CT/TUC+: control diet with Tucum‐do‐Cerrado; HF/TUC‐: high‐fat diet; HF/TUC+: high‐fat diet with Tucum‐do‐Cerrado; TUC‐: without Tucum‐do‐Cerrado; TUC+: with Tucum‐do‐Cerrado.

Figure 2A illustrates the effects of Tucum‐do‐Cerrado on adipose tissue morphology. In BAT, the high‐fat diet showed a tendency to promote a twofold increase in adipocyte area (HF/TUC‐ = 1002.64 and CT/TUC‐ = 509.24 µm^2^, *p* = 0.055), while the addition of Tucum‐do‐Cerrado to the high‐fat diet (HF/TUC+) reduced adipocyte size by 2.2‐fold (HF/TUC+ = 461.33 and HF/TUC‐ = 1002.64 µm^2^, *p* = 0.034; Figure 2A,B). A similar profile was obtained in the iWAT, where the high‐fat diet increased the adipocyte area 1.5‐fold (high‐fat = 3,791.08 µm^2^ and control = 2,462.59 µm^2^, *p* < 0.001), whereas Tucum‐do‐Cerrado reduced adipocyte area 1.3‐fold (TUC+ = 2,733.64 µm^2^ and TUC‐ = 3,520.03 µm^2^, *p* = 0.017), regardless of the diet type (Figure 2A,B).

Contrary to the other adipose tissues, in eWAT, only a diet effect was observed, with a 1.3‐fold increase in adipocyte area in the high‐fat group compared to the control group (high‐fat = 3,145.80 and control = 2,474.24 µm^2^, *p* = 0.029; Figure 2A,B).

### 3.3 Adipocyte Morphological Type and Immunohistochemistry

**FIGURE 3:**
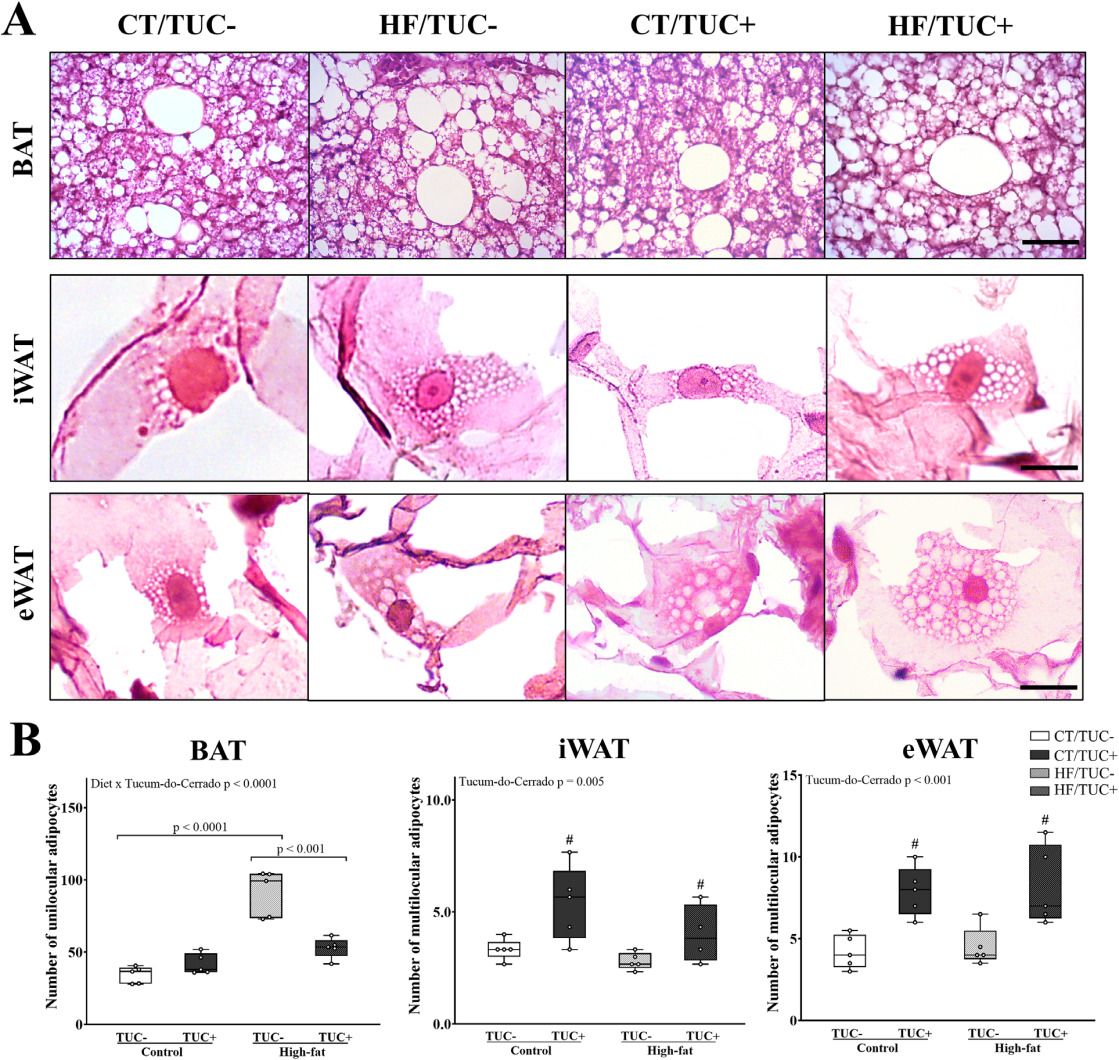
Unilocular and Multilocular adipocytes of adipose tissue from rats. (A) Representative histological images of unilocular adipocytes in brown adipose tissue (BAT) and multilocular adipocytes in inguinal (iWAT) and epididymal (eWAT) adipose tissues. Stain: hematoxylin and eosin. Scale bar: 10 µm (BAT) and 5 µm (iWAT and eWAT); (B) Quantification of unilocular adipocytes in BAT and multilocular adipocytes in iWAT and eWAT. Values are presented as box plots with whiskers from minimum to maximum (*n* = 5/group). *p < 0.05 effect of diet; # *p* < 0.05 effect of Tucum‐do‐Cerrado. CT/TUC‐: control diet; CT/TUC+: control diet with Tucum‐do‐Cerrado; HF/TUC‐: high‐fat diet; HF/TUC+: high‐fat diet with Tucum‐do‐Cerrado; TUC‐: without Tucum‐do‐Cerrado; TUC+: with Tucum‐do‐Cerrado.

In BAT, a diet and Tucum‐do‐Cerrado interaction was observed for the unilocular adipocyte count (Figure 3B). The high‐fat diet markedly increased the unilocular adipocyte count (*p* < 0.0001), whereas HF/TUC+ decreased the unilocular adipocyte count compared with that in the HF/TUC‐ group (*p* < 0.001).

Morphological analysis of iWAT and eWAT revealed a Tucum‐do‐Cerrado effect on the number of multilocular adipocytes, whereas its consumption increased their number, regardless of the diet type (*p* = 0.005 and *p* < 0.001, respectively; Figure 3B).

**FIGURE 4:**
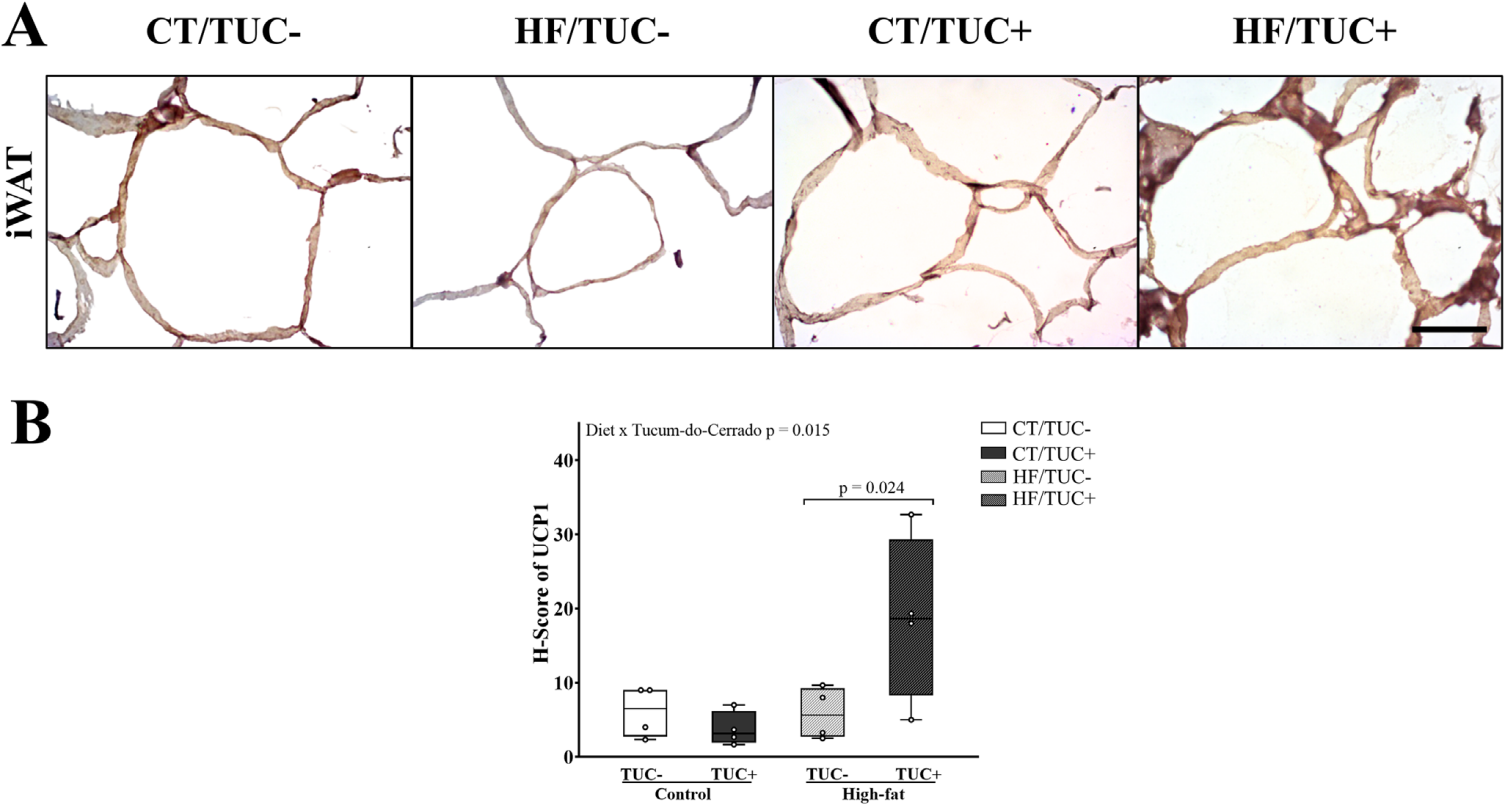
UCP1 immunostaining in iWAT (H‐score) from rats. (A) Adipocyte immunostained with UCP1 in inguinal adipose tissue (iWAT). Counterstain: hematoxylin. Scale bar = 5 µm. (B) Histoscore (H‐score) quantification of UCP1 immunostaining. Values are presented as box plots with whiskers from minimum to maximum (*n* = 5/group). *p < 0.05 effect of diet; # *p* < 0.05 effect of Tucum‐do‐Cerrado. CT/TUC‐: control diet; CT/TUC+: control diet with Tucum‐do‐Cerrado; HF/TUC‐: high‐fat diet; HF/TUC+: high‐fat diet with Tucum‐do‐Cerrado; TUC‐: without Tucum‐do‐Cerrado; TUC+: with Tucum‐do‐Cerrado.

In iWAT immunohistochemistry (Figure 4A,B), H‐score analysis of anti‐UCP1 staining revealed a diet and Tucum‐do‐Cerrado interaction. The HF/TUC+ diet increased the score compared to the HF/TUC‐ (*p* = 0.024; Figure 4B).

### 3.4 Hepatic Steatosis Score

**FIGURE 5:**
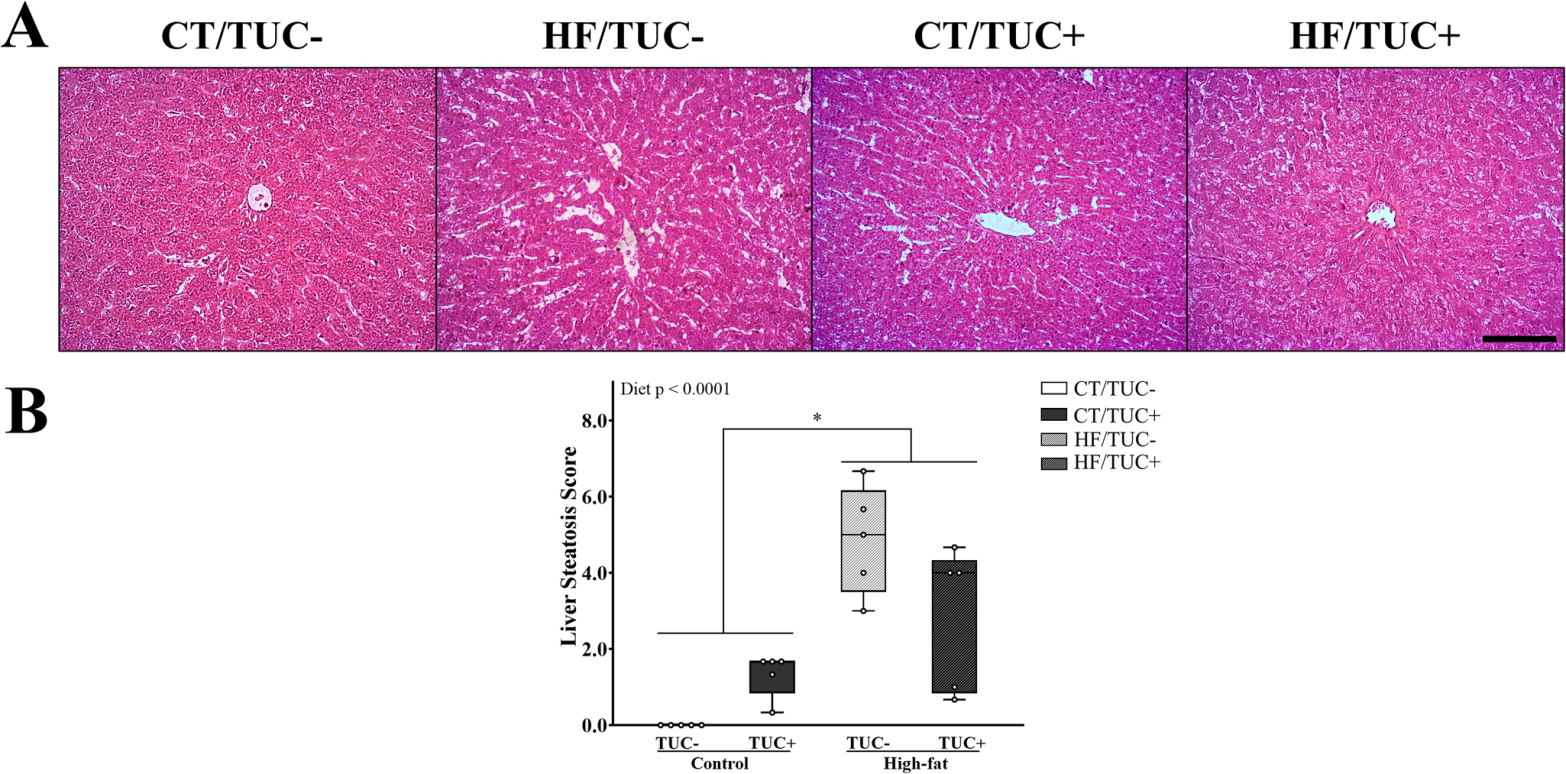
Liver histology from rats. (A) Histological sections of liver tissue. Stain: hematoxylin and eosin. Scale bar: 20 µm. (B) Quantification of liver steatosis score. Values are presented as box plots with whiskers from minimum to maximum (*n* = 5/group). *p < 0.05 effect of diet; # *p* < 0.05 effect of Tucum‐do‐Cerrado. CT/TUC‐: control diet; CT/TUC+: control diet with Tucum‐do‐Cerrado; HF/TUC‐: high‐fat diet; HF/TUC+: high‐fat diet with Tucum‐do‐Cerrado; TUC‐: without Tucum‐do‐Cerrado; TUC+: with Tucum‐do‐Cerrado.

The HF/TUC‐ diet resulted in a marked increase in the liver steatosis score relative to the CT/TUC‐ diet (*p* < 0.001; Figure 5B), whereas Tucum‐do‐Cerrado consumption did not affect this parameter.

### 3.5 mRNA Levels of Lipid Metabolism Genes in Adipose Tissues

**FIGURE 6:**
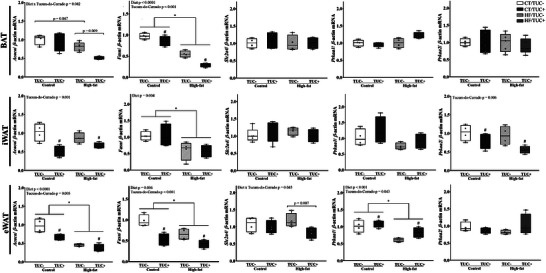
mRNA levels of genes involved in lipid metabolism of rats. Values are presented as box plots with whiskers from minimum to maximum (*n* = 6/group) in brown (BAT), inguinal (iWAT), and epididymal (eWAT) adipose tissues. *p < 0.05 effect of diet; # *p* < 0.05 effect of Tucum‐do‐Cerrado. CT/TUC‐: control diet; CT/TUC+: control diet with Tucum‐do‐Cerrado; HF/TUC‐: high‐fat diet; HF/TUC+: high‐fat diet with Tucum‐do‐Cerrado; TUC‐: without Tucum‐do‐Cerrado; TUC+: with Tucum‐do‐Cerrado.

Figure 6 shows the expression of lipid metabolism‐related genes in BAT, iWAT, and eWAT. In BAT, a diet and Tucum‐do‐Cerrado interaction was obtained for *Acaca* expression; the *Acaca* mRNA levels were downregulated in the HF/TUC‐ compared to the CT/TUC‐ (*p* = 0.047), and in the HF/TUC+ compared to the HF/TUC‐ (*p* = 0.009). The high‐fat diet and Tucum‐do‐Cerrado consumption, regardless of the diet type, decreased *Fasn* mRNA levels (*p* < 0.0001 and *p* < 0.001, respectively).

In the iWAT, Tucum‐do‐Cerrado consumption decreased the mRNA levels of *Acaca* and *Prkaa2*, regardless of the diet type (*p* = 0.001 and 0.006, respectively; Figure 6). Diet type also affected *Fasn* transcription, with lower mRNA levels in the high‐fat diet than in the control diet (*p* = 0.004).

Finally, in eWAT, *Acaca and Fasn* mRNA levels decreased with the high‐fat diet compared to the control diet (*p* < 0.0001 and 0.006, respectively) and with Tucum‐do‐Cerrado consumption, regardless of the diet type (*p* = 0.005 and *p* < 0.001; Figure 6). Additionally, the *Slc2a4* gene was downregulated by HF/TUC+ in relation to HF/TUC‐ (*p* = 0.007), whereas *Prkaa1* mRNA levels decreased with the high‐fat diet and increased with Tucum‐do‐Cerrado consumption (*p* < 0.001 and *p* = 0.043, respectively).

### 3.6 mRNA Levels of Thermogenic Markers in Different Adipose Depots

**FIGURE 7:**
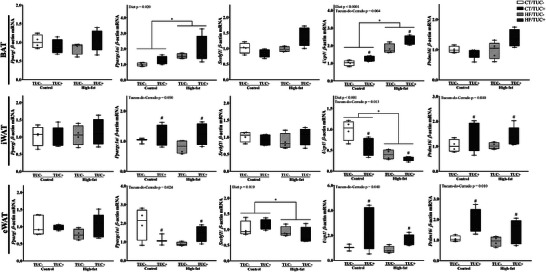
mRNA levels of thermogenic‐related genes from rats. Values are presented as box plots with whiskers from minimum to maximum (*n* = 6/group) in brown (BAT), inguinal (iWAT), and epididymal (eWAT) adipose tissues. *p < 0.05 effect of diet; # *p* < 0.05 effect of Tucum‐do‐Cerrado. CT/TUC‐: control diet; CT/TUC+: control with Tucum‐do‐Cerrado; HF/TUC‐: high‐fat diet; HF/TUC+: high‐fat with Tucum‐do‐Cerrado; TUC‐: without Tucum‐do‐Cerrado; TUC+: with Tucum‐do‐Cerrado.

The analysis of genes involved in thermogenesis is shown in Figure 7. In BAT, the high‐fat diet upregulated *Ppargc1a* and *Ucp1* mRNA expression levels (*p* = 0.020 and *p* < 0.0001, respectively). Additionally, Tucum‐do‐Cerrado consumption increased *Ucp1* expression, regardless of the diet type (*p* = 0.004).

In iWAT, Tucum‐do‐Cerrado consumption upregulated *Ppargc1a* and *Prdm16* mRNA levels, while downregulating *Ucp1* mRNA levels, regardless of the diet type (*p* = 0.005, 0.040, and 0.013, respectively; Figure 7). Furthermore, in the iWAT, *Ucp1* mRNA levels decreased in the high‐fat group compared to the control group (*p* < 0.001).

Considering eWAT, Tucum‐do‐Cerrado consumption upregulated *Ppargc1a*, *Ucp1*, and *Prdm16* mRNA levels, regardless of the diet type (*p* = 0.024, 0.040, and 0.010, respectively; Figure 7). The high‐fat diet downregulated *Srebf1* mRNA levels compared to the control diet (*p* = 0.019).

### 3.7 mRNA Levels of Genes Involved in Angiogenesis and Inflammation

**FIGURE 8:**
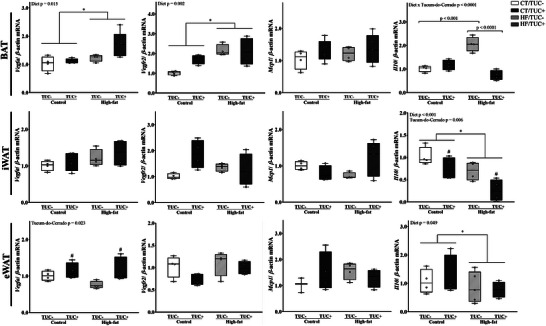
mRNA levels of angiogenesis and inflammation‐related genes. Values are presented as box plots with whiskers from minimum to maximum (*n* = 6/group) in brown (BAT), inguinal (iWAT), and epididymal (eWAT) adipose tissues. *p < 0.05 effect of diet; # *p* < 0.05 effect of Tucum‐do‐Cerrado. CT/TUC‐: control diet; CT/TUC+: control with Tucum‐do‐Cerrado; HF/TUC‐: high‐fat diet; HF/TUC+: high‐fat with Tucum‐do‐Cerrado; TUC‐: without Tucum‐do‐Cerrado; TUC+: with Tucum‐do‐Cerrado.

Regarding genes related to angiogenesis (Figure 8), in BAT, *Vegfa* and *Vegfr2* mRNA levels were upregulated by the high‐fat diet (*p* = 0.015 and 0.002, respectively). In eWAT, Tucum‐do‐Cerrado consumption increased *Vegfa* mRNA levels (*p* = 0.023).

For inflammatory genes in BAT, HF/TUC‐ upregulated *Il10* mRNA levels in relation to CT/TUC‐ (*p* < 0.001), whereas HF/TUC+ downregulated its levels compared to HF/TUC‐ (*p* < 0.0001; Figure 8). A different response was obtained in the iWAT, where both the high‐fat diet and Tucum‐do‐Cerrado consumption downregulated *Il10* mRNA levels (*p* < 0.001 and p = 0.006, respectively).

### 3.8 Oxidative Stress Markers

**FIGURE 9:**
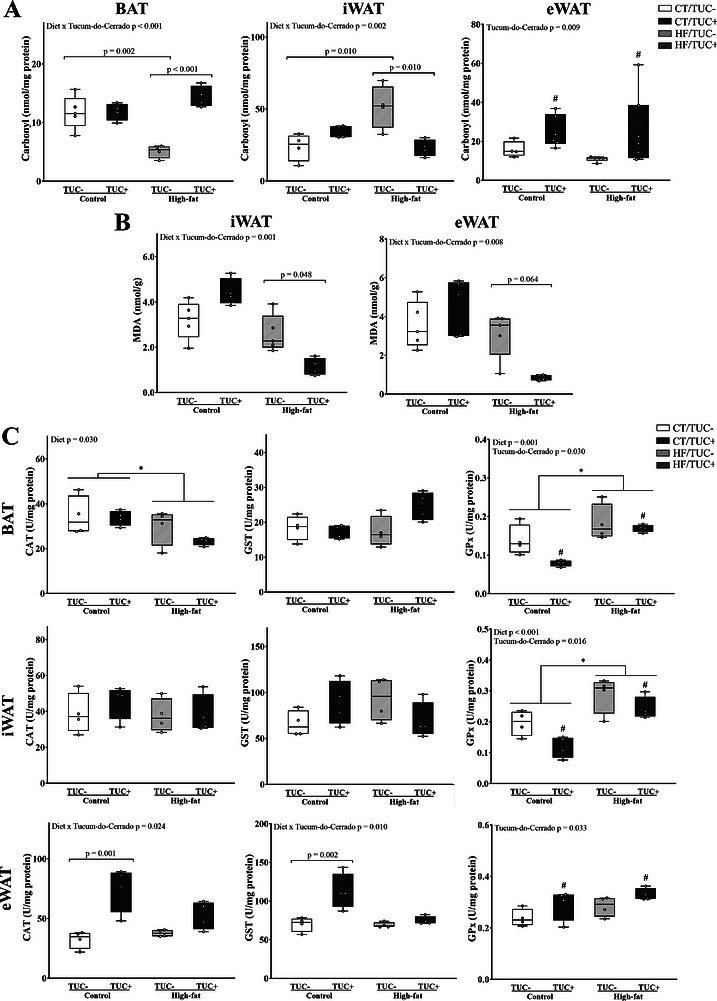
Oxidative stress markers and antioxidant enzyme activities in rats. (A) Carbonyl protein content. (B) Malondialdehyde (MDA) levels. (C) Activity of antioxidant enzymes. Values are presented as box plots with whiskers from minimum to maximum (*n* = 5/group oxidative damage and 4/group antioxidant enzymes) in brown (BAT), inguinal (iWAT), and epididymal (eWAT) adipose tissues. *p < 0.05 effect of diet; # *p* < 0.05 effect of Tucum‐do‐Cerrado. CT/TUC‐: control diet; CT/TUC+: control with Tucum‐do‐Cerrado; HF/TUC‐: high‐fat diet; HF/TUC+: high‐fat with Tucum‐do‐Cerrado; TUC‐: without Tucum‐do‐Cerrado; TUC+: with Tucum‐do‐Cerrado; CAT: catalase; GST: Glutathione S‐transferase; GPx: Glutathione peroxidase; MDA: malondialdehyde.

Carbonyl and malondialdehyde concentrations are shown in Figure 9. In BAT, the HF/TUC‐ diet reduced carbonyl content in relation to CT/TUC‐ (*p* = 0.002), whereas in iWAT, this increased (*p* = 0.010; Figure 9A). The HF/TUC+ diet increased carbonyl content compared to the HF/TUC‐ in BAT (*p* < 0.001), while in iWAT it decreased (*p* = 0.010). In eWAT, increased carbonyl content was observed with Tucum‐do‐Cerrado consumption (*p* = 0.009).

Regarding lipid peroxidation (Figure 9B), in iWAT and eWAT, the HF/TUC+ group, the MDA concentration decreased compared to the HF/TUC‐ group (*p* = 0.048 and 0.064, respectively).

### 3.9 Enzymatic Antioxidant Response

The activities of the antioxidant enzymes are shown in Figure 9C. In BAT, catalase activity was reduced in rats fed the high‐fat diet compared to those fed the control diet (*p* = 0.030). In eWAT, CT/TUC+ promoted a 2.3‐fold increase in CAT activity compared to that in CT/TUC‐ (*p* = 0.001).

Regarding GST activity, the CT/TUC+ group had increased activity in eWAT compared to the CT/TUC‐ group (*p* = 0.002).

Finally, in BAT and iWAT, the high‐fat diet increased GPx activity compared to the control diet (*p* = 0.001 and *p* < 0.001, respectively). However, Tucum‐do‐Cerrado consumption decreased GPx activity in BAT and iWAT (*p* = 0.030 and 0.016, respectively) while increasing activity in eWAT (*p* = 0.033).

## 4 Discussion

Tucum‐do‐Cerrado consumption promoted a healthier expansion of adipose tissue in a high‐fat diet‐induced obesity model, preventing adipocyte hypertrophy and promoting an increase in multilocular lipid droplets. However, these effects were distinct across different adipose depots.

The high‐fat diet consumption resulted in adipose tissue dysfunction, evidenced by increased body weight, hypertrophy of BAT and iWAT, high triglyceride levels, and steatosis scores in the liver. However, the reduction in food intake, the downregulation of genes involved in fatty acid synthesis and oxidation (*Fasn* in all tissues and *Acaca* in BAT and iWAT), suggests an attempt of HF/TUC‐ treated rats to regulate excessive body weight. These results may explain the non‐effect of Tucum‐do‐Cerrado on body weight gain of HF/TUC+ rats during the treatment period. Furthermore, the high‐fat diet upregulated the expression of thermogenic‐related genes (*Ppargc1a*, *Ucp1*, *Vegfa*, and *Vegfr2* in BAT), supporting this hypothesis. This response is well documented in the literature; rats were categorized as prone and resistant to obesity development upon high‐fat diet treatment based on their body weight gain [32]. Previous findings have demonstrated that a high‐fat diet initially induces the expression of thermogenic genes in BAT as an adaptive response to maintain energy homeostasis [33].

The reduction in adipose tissue hypertrophy promoted by Tucum‐do‐Cerrado was not accompanied by lower body weight gain. One explanation for these findings would be that Tucum‐do‐Cerrado may have induced lipolysis and/or inhibited fatty acid uptake in adipocytes. The mRNA gene analysis revealed the downregulation of *Acaca* in both BAT and iWAT and of *Fasn* in BAT, which are considered lipogenic regulators. Therefore, these data suggest that malonyl‐CoA levels were reduced, and consequently, fatty acid synthesis was inhibited in favor of oxidation. The tendency of Tucum‐do‐Cerrado to decrease serum triglycerides (TUC+: 85.3 (68.9–101.6) and TUC‐: 107.3 (91.3–123.3) mg/dL; *p* = 0.052) associated with no effect on their levels in the liver, reinforces the hypothesis that oxidation was stimulated rather than lipid uptake was inhibited. Notably, these findings are consistent with studies on other polyphenol‐rich fruits, where metabolic improvements in adipose tissue occurred independently of changes in total body weight. For instance, without alterations in body weight, Gibert‐Ramos et al. [34] observed that sweet cherry supplementation significantly downregulated *Fasn* and *Acaca* expression and reduced adipocyte area. Similarly, Dziadek et al. [35] reported anti‐lipogenic effects from freeze‐dried fruits. These authors attributed such effects to the anthocyanin content, a compound also present in Tucum‐do‐Cerrado fruit. The increase in hepatic total cholesterol levels promoted by Tucum‐do‐Cerrado consumption may be related to the upregulation of LDL activity promoted by polyphenols. According to Zern and Fernandez [36], grape polyphenols initially promote a decrease in intracellular cholesterol levels. Subsequently, in a feedback response, hepatocytes upregulate LDL receptor mRNA and activity to compensate for the deficiency, leading to an increase in hepatic cholesterol concentration. However, this does not appear to be a potential side effect, as the hepatic steatosis score remained unchanged with Tucum‐do‐Cerrado consumption.

Another mechanism that could explain the attenuation of adipocyte hypertrophy by Tucum‐do‐Cerrado consumption is the stimulation of adipogenesis. Among the genes involved in the regulation of adipogenesis in adipose tissue, PPARγ is the major regulator of adipocyte differentiation [37]. Consistent with this appointment, the upregulation of *Ppargc1a* mRNA levels in iWAT and downregulation of *Acaca* mRNA levels suggest that Tucum‐do‐Cerrado counteracts adipocyte hypertrophy by stimulation of both adipogenesis and lipolysis, rather than by reducing fatty acid uptake. Munkong et al. [38] showed that the extract of *Elaeagnus latifolia* fruit activates the transcription factor, *Pparg*, resulting in smaller adipocyte size, reduced inflammation, and oxidative stress in a high‐fat diet induced model. The authors suggested that bioactive compounds present in *Elaeagnus latifolia* fruit stimulate adipogenesis, improving adipocyte function in obese mice. Similarly, the administration of grape seed proanthocyanidin extract induces adipogenesis of WAT through the upregulation of *Pparg* [39].

Although the stimulation of adipogenesis is related to a potential increase in adipose tissue mass [37], the apparent pro‐adipogenic effect of Tucum‐do‐Cerrado can be considered a beneficial factor for metabolic health, since this response occurs without an increase in total body weight. Some polyphenols, such as resveratrol and quercetin, have been shown to induce healthier adipose expansion by reducing adipocyte size, independent of changes in total body weight [40, 41]. Supporting this hypothesis, mRNA levels of the thermogenic transcription co‐regulator PRDM16, as well as the protein content of UCP1, increased with the consumption of Tucum‐do‐Cerrado in iWAT, and in BAT, *Ucp1* mRNA increased. Small adipocytes can counteract hypoxia associated with hypertrophic adipocytes, thereby inhibiting tissue inflammation [42]. The increase in UCP1 protein in iWAT, despite decreased mRNA levels, may be related to the protein's longer half‐life compared to mRNA. Gospodarska et al. [43], observed that UCP1 protein declined more slowly than mRNA when the cold stimulus ceased, while *Ucp1* mRNA showed a precipitous decline. The authors suggested that proteins embedded in the mitochondrial membrane are more stable than mRNA. Similarly, in eWAT, Tucum‐do‐Cerrado upregulated *Ppargc1a, Ucp1, Prdm16, Vegfa*, and *Prkaa1*, without affecting adipocyte hypertrophy. As observed in our study, diet‐induced obese mice supplemented with 15% of Jaboticaba peel and seed powder showed increased of *Ucp1* and *Prdm16* levels in WAT and BAT. Furthermore, jaboticaba reduced adipocyte size in WAT [11].

Excess adipose tissue accumulation is characterized by local inflammation and BAT dysfunction [44]. In the present study, the increase in the anti‐inflammatory cytokine *Il10* mRNA by the HF/TUC‐ diet in BAT suggests a counterregulatory effect on the pro‐inflammatory environment promoted by chronic lipid overload. Nga et al. [45] showed that impairment of BAT function (reduction of UCP1 levels) in mice housed under thermoneutrality conditions was accompanied by an increased expression of pro‐inflammatory cytokines and the anti‐inflammatory IL‐10. The authors suggested that the pronounced increase in IL‐10 expression by BAT is an adaptive response to attenuate tissue dysfunction. These results corroborate the hypothesis that under stress conditions, BAT upregulates IL‐10 expression to counteract the damage promoted by inflammation.

Rajbhandari et al. [46] reported that marker genes of adipose tissue browning and UCP‐1 protein were increased in iWAT of Il‐10^−^/^−^ mice. The authors suggested that the loss of IL‐10 increases energy expenditure and protects against high‐fat diet‐induced obesity. Considering that the reduction in IL‐10 levels in adipose tissue induces thermogenic expression, we suggest that the reduction in adipocyte hypertrophy and the improvement in metabolic dysfunction promoted by the consumption of Tucum‐do‐Cerrado are associated with the downregulation of *Il10* mRNA levels in BAT and iWAT. Corroborating this hypothesis, in iWAT, Tucum‐do‐Cerrado increased UCP1 protein levels in HF/TUC+ diet‐treated rats and *Prdm16*, regardless of the diet type.

Adipose tissue hypertrophy and inflammation in obesity are associated with the induction of oxidative stress [47]. The high availability of fatty acids in the iWAT of high‐fat rats affected redox status; the carbonyl increase was accompanied by an increase in GPx activity, suggesting an attempt to mitigate oxidative response and tissue dysfunction. Although GPx activity also increased in BAT, protein oxidation decreased in this tissue. The lower accumulation of fatty acids in BAT than in WAT may have attenuated the oxidative response, and thus, the increase in GPx activity was sufficient to decrease protein oxidation.

Tucum‐do‐Cerrado peel has a high phenolic concentration in the methanolic extract [13]. Therefore, we hypothesized that the hydrophobic nature of some phenolics in Tucum‐do‐Cerrado may protect adipocytes against oxidation. Overall, Tucum‐do‐Cerrado improved the antioxidant capacity of iWAT and eWAT of high‐fat diet‐treated rats, reducing the oxidative stress caused by excessive consumption of fatty acids. MDA levels were decreased in the HF/TUC+ group compared to the HF/TUC‐ in both WAT, as was the carbonyl content in iWAT. Additionally, the reduced GPx activity in iWAT may be attributed to the high polyphenol content of Tucum‐do‐Cerrado, which exhibits high antioxidant activity [13, 18], thereby reducing the demand for enzymatic antioxidant activity. Improving the non‐enzymatic antioxidant capacity of tissues through the consumption of dietary antioxidants decreases oxidative tissue status and consequently modulates enzymatic activity [48]. Together, these data indicate that Tucum‐do‐Cerrado's effects on adipose tissues may be attributed to its polyphenol content and consequent high antioxidant capacity. Dietary polyphenols, such as resveratrol, quercetin, and apple‐derived polyphenols, have also been shown to promote healthier characteristics in white adipose tissue [49, 50]. *Euterpe oleracea* Mart. (açaí) seed extract, rich in catechin, epicatechin, and proanthocyanidins, reduced lipid peroxidation markers (8‐Isoprostane and MDA) and increased superoxide dismutase and GPx activity in the visceral adipose tissue [51], reinforcing our hypothesis that fruits rich in polyphenols exert beneficial effects in redox response on obesity‐related white adipose tissue.

## 5 Conclusions

Tucum‐do‐Cerrado consumption promoted healthier expansion of adipose tissue in a high‐fat diet‐induced obesity model by stimulating lipid oxidation and adipogenesis in brown and inguinal adipose tissues. The effects of Tucum‐do‐Cerrado on adipose tissues may be attributed to its polyphenol content and consequent improvement in antioxidant capacity and inflammatory response.

## Author Contributions

**Marilia Hermes Cavalcanti**:, **Sandra Fernandes Arruda**: Conceptualization. **Marilia Hermes Cavalcanti**:, **Sandra Fernandes Arruda**: Data curation. **Marilia Hermes Cavalcanti**:, **Sandra Fernandes Arruda**: Formal analysis. **Marilia Hermes Cavalcanti**:, **Sandra Fernandes Arruda**: Funding acquisition. **Marilia Hermes Cavalcanti**: Investigation. **Marilia Hermes Cavalcanti**:, **Sandra Fernandes Arruda**:, **Amílcar Sabino Damazo**: Methodology. **Sandra Fernandes Arruda**: Project administration. **Sandra Fernandes Arruda**:, **Amílcar Sabino Damazo**: Resources. **Marilia Hermes Cavalcanti**:, **Sandra Fernandes Arruda**:, **Amílcar Sabino Damazo**: Software. **Sandra Fernandes Arruda**: Supervision. **Marilia Hermes Cavalcanti**:, **Sandra Fernandes Arruda**:, **Amílcar Sabino Damazo**: Validation. **Marilia Hermes Cavalcanti**:, **Sandra Fernandes Arruda**:, **Amílcar Sabino Damazo**: Visualization. **Marilia Hermes Cavalcanti**:, **Sandra Fernandes Arruda**:, **Amílcar Sabino Damazo**: Writing – original draft. **Marilia Hermes Cavalcanti**:, **Sandra Fernandes Arruda**:, Writing – review & editing.

## Conflicts of Interest

The authors declare no conflicts of interest.

## Supporting information




**Supporting File**: mnfr70498‐sup‐0001‐SupMat.docx.

## Data Availability

The data that support the findings of this study are available on request from the corresponding author. The data are not publicly available due to privacy or ethical restrictions.
