# Supplementary material for: Tucum‐do‐Cerrado (Bactris setosa Mart.) Consumption Promoted a Healthier Expansion of Adipose Tissue in High‐Fat Diet‐Induced Obesity Rats
Source: Mol Nutr Food Res. 2026 Jun 30;70(13):e70498. doi: 10.1002/mnfr.70498 (PMC13317759; doi:10.1002/mnfr.70498)
Supplement: Supplementary file 1 — Supporting File: mnfr70498‐sup‐0001‐SupMat.docx. [file MNFR-70-e70498-s001.docx]

**Table S1.** Ingredients and composition of the experimental diets.

| **Ingredient (g)** |  | **CT/TUC-** | **CT/TUC+** | **HF/TUC-** | **HF/TUC+** |
| --- | --- | --- | --- | --- | --- |
| Corn starch |  | 397.5 | 369.686 | 184.1 | 155.8 |
| Casein |  | 200 | 198.3 | 264.9 | 263.1 |
| Dextrinized starch |  | 132 | 132 | 60.8 | 60.8 |
| Sucrose |  | 100 | 100 | 46.6 | 46.6 |
| Soybean oil |  | 70 | 69.7 | 35.7 | 35.4 |
| Cellulose |  | 50 | 47.5 | 50.9 | 48.4 |
| Mixed mineral |  | 35 | 35 | 35.7 | 35.7 |
| Mixed vitamin |  | 10 | 10 | 10.2 | 10.2 |
| L-cystine |  | 3.0 | 3.0 | 3.1 | 3.1 |
| Choline bitartrate |  | 2.5 | 2.5 | 2.5 | 2.6 |
| Tertbutyl |  | 0.014 | 0.014 | 0.014 | 0.014 |
| Lard |  | - | - | 305.6 | 305.6 |
| Tucum-do-Cerrado | (g) | - | 150 | - | 150 |
| (*Bactris setosa* Mart) | Carbohydrate | - | 27.8 | - | 28.3 |
|  | Protein | - | 1.7 | - | 1.7 |
|  | Lipid | - | 0.3 | - | 0.3 |
|  | Fiber | - | 2.5 | - | 2.6 |
| Total flavanols (717 mg catechin /100g), anthocyanins (83 mg/100g), yellow flavonoids (42 mg/100g), and vitamin C (78 mg/100g).^1^ | | | | | |
| Energy | % |  |  |  |  |
|  | Carbohydrate | 64 | 64 | 22 | 22 |
|  | Protein | 20 | 20 | 20 | 20 |
|  | Fat | 16 | 16 | 58 | 58 |

CT/TUC-: rats fed with control diet (AIN-93G); CT/TUC+: rats fed with control diet + Tucum-do-Cerrado; HF/TUC-: rats fed with high-fat diet; HF/TUC+: rats fed with high-fat diet + Tucum-do-Cerrado.^1^ Siqueira et al., 2013.

**Table S2.** Primer sequences used for qPCR.

| **Gene** | **Primer sequences (5’- 3’)** | **N° access GenBank** | | **Reference** |
| --- | --- | --- | --- | --- |
| *Acaca*  (ACC) | GATGATCAAGGCCAGCTTGT (F)  CAGGCTACCATGCCAATCTC (R) | NM_022193.1 | | Moreno-Fernández et al. [1] |
| *Fasn*  (FAS) | GGCCACCTCAGTCCTGTTAT (F)  AGGGTCCAGCTAGAGGGTACA (R) | M76767.1 | | Moreno-Fernández et al. [1] |
| *Slc2a4*  (GLUT4) | TTGCAGTGCCTGAGTCTTCTT (F)  CCAGTCACTCGCTGCTGA (R) | NM_012751.1 | | Snoussi et al. [2] |
| *Prkaa1*  (AMPK-ɑ1) | GAAGTCAAAGCCGACCCAAT (F)  AGGGTTCTTCCTTCGCACAC (R) | NM_019142 | | Shi et al. [3] |
| *Prkaa2*  (AMPK-ɑ2) | ATGATGAGGTGGTGGAGCAG (F)  GTGAATGGTTCTCGGCTGTG (R) | NM_023991 | | Shi et al. [3] |
| *Pparg* | CTGAAGCTCCAAGAATACCA (F)  TCCCCACAGACTCGGCACTC (R) | NM_013124.3 | | Lanzi et al. [4] |
| *Ppargc1*α  (PGC-1α) | AAAAGCTTGACTGGCGTCAT (F)  TCAGGAAGATCTGGGCAAAG (R) | NM_031347.1 | | Lanzi et al. [4] |
| *Srebf1*  (SREBP-1c) | ACAAGATTGTGGAGCTCAAGG (F)  TGCGCAAGACAGCAGATTTA (R) | NM_001276707.1 | | Coronado-Cáceres et al. [5] |
| *Ucp1* | TGGCGTGGCGGTATTCAT (F)  GGCTTTGTGCTTGCATTCTG (R) | NM_012682.2 | | Shen et al. [6] |
| *Prdm16* | TTGGTGCATGTGAAAGAAGG (F)  CCTCAGGCTTGAGCTCCTC (R) | XM_039111362.1 | | Lanzi et al. [4] |
| *Vegfa* | AATGATGAAGCCCTGGAGTG (F)  ATGCTGCAGGAAGCTCATCT (R) | NM_031836.3 | | Lanzi et al. [4] |
| *Vegfr2* | TAGCACGACAGAGACTGTGAGG (F)  TGAGGTGAGAGAGATGGGTAGG (R) | | NM_013062.2 | Lanzi et al. [4] |
| *Il10* | AGTGGAGCAGGTGAAGAATGA (F)  TCATGGCCTTGTAGACACCTT (R) | NM_012854.2 | | Coronado-Cáceres et al. [5] |
| *Mcp1* | TGCAGGTCTCTGTCACGCTTC (F)  TTCTCCAGCCGACTCATTGG (R) | NM_031530.1 | | Lanzi et al. [4] |
| *Actβ*  (Actin β) | GTCGTACCACTGGCATTGTG (F)  CTCTCAGCTGTGGTGGTGAA (R) | NM_031144 | | Wang et al. [7] |

(F): forward primer; (R): reverse primer. *Acaca*: acetyl-CoA carboxyl*; Fasn*: fatty acid synthase; *Slc2a4*: solute carrier family 2 member 4; *Prkaa1*: protein kinase AMP-activated catalytic subunit alpha 1; *Prkaa2*: protein kinase AMP-activated catalytic subunit alpha 2; *Pparg*: peroxisome proliferator-activated receptor gamma; *Ppargc1a*: peroxisome proliferator-activated receptor gamma coactivator 1α; *Srebf1*: sterol regulatory element-binding transcription factor 1; *Ucp1*: uncoupling protein 1; *Prdm16*: PR domain-containing protein 16; *Vegfa*: vascular endothelial growth factor A; *Vegfr2*: vascular endothelial growth factor receptor 2; *Il10*: interleukin 10; *Mcp1*: monocyte chemotactic protein-1; *Actβ*: beta-actin.

**References**

[1] S. Moreno-Fernández, M. Garcés-Rimón, J. A. Uranga, J. Astier, J. F. Landrier, M. Miguel, *Food Funct.* **2018**, *9*, 6599. DOI: 10.1039/C8FO01754A.

[2] C. Snoussi, R. Ducroc, M. H. Hamdaoui, K. Dhaouadi, H. Abaidi, F. Cluzeaud, C. Nazaret, M. Le Gall, A. Bado, *J. Nutr. Biochem.* **2014**, *25*, 557. DOI: 10.1016/j.jnutbio.2014.01.006.

[3] X. X. Shi, B. S. Yin, P. Yang, H. Chen, X. Li, L. X. Su, H. G. Fan, H. B. Wang, *PLoS One* **2016**, *11*, e0153169. DOI: 10.1371/journal.pone.0153169.

[4] C. R. Lanzi, D. J. Perdicaro, M. S. Landa, A. Fontana, A. Antoniolli, R. M. Miatello, P. I. Oteiza, M. A. Vazquez Prieto, *J. Nutr. Biochem.* **2018**, *56*, 224. DOI: 10.1016/j.jnutbio.2018.03.001.

[5] L. J. Coronado-Cáceres, G. Rabadán-Chávez, L. Quevedo-Corona, B. Hernández-Ledesma, A. M. Garcia, L. Mojica, E. Lugo-Cervantes, *J. Funct. Foods* **2019**, *62*, 103519. DOI: 10.1016/j.jff.2019.103519.

[6] H. H. Shen, S. Y. Huang, C. W. Kung, S. Y. Chen, Y. F. Chen, P. Y. Cheng, K. K. Lam, Y. M. Lee, J. Nutr. Biochem. **2019**, *67*, 111. DOI: 10.1016/j.jnutbio.2019.02.001.

[7] Q. Wang, F. Du, Z. M. Qian, X. H. Ge, L. Zhu, W. H. Yung, *Endocrinology* **2008**, *149*, 3920. DOI: 10.1210/en.2007-1626.
